# Development and Validation of a Preoperative Nomogram for Predicting Benign and Malignant Gallbladder Polypoid Lesions

**DOI:** 10.3389/fonc.2022.800449

**Published:** 2022-03-25

**Authors:** Shuai Han, Yu Liu, Xiaohang Li, Xiao Jiang, Baifeng Li, Chengshuo Zhang, Jialin Zhang

**Affiliations:** ^1^Department of Hepatobiliary Surgery, The First Hospital of China Medical University, Shenyang, China; ^2^Department of Radiology, The First Hospital of China Medical University, Shenyang, China; ^3^Department of Endocrinology and Metabolism, The Second Hospital of Dalian Medical University, Dalian, China

**Keywords:** gallbladder polypoid lesions, radiomics, nomogram, computed tomography, risk factors

## Abstract

**Purpose:**

The purpose of this study was to develop and validate a preoperative nomogram of differentiating benign and malignant gallbladder polypoid lesions (GPs) combining clinical and radiomics features.

**Methods:**

The clinical and imaging data of 195 GPs patients which were confirmed by pathology from April 2014 to May 2021 were reviewed. All patients were randomly divided into the training and testing cohorts. Radiomics features based on 3 sequences of contrast-enhanced computed tomography were extracted by the Pyradiomics package in python, and the nomogram further combined with clinical parameters was established by multiple logistic regression. The performance of the nomogram was evaluated by discrimination and calibration.

**Results:**

Among 195 GPs patients, 132 patients were pathologically benign, and 63 patients were malignant. To differentiate benign and malignant GPs, the combined model achieved an area under the curve (AUC) of 0.950 as compared to the radiomics model and clinical model with AUC of 0.929 and 0.925 in the training cohort, respectively. Further validation showed that the combined model contributes to better sensitivity and specificity in the training and testing cohorts by the same cutoff value, although the clinical model had an AUC of 0.943, which was higher than 0.942 of the combined model in the testing cohort.

**Conclusion:**

This study develops a nomogram based on the clinical and radiomics features for the highly effective differentiation and prediction of benign and malignant GPs before surgery.

## Introduction

Gallbladder polypoid lesions (GPs), as a common gallbladder disease, represent a wide spectrum of lesions that protrude inward from the wall of the gallbladder. In past decades, the prevalence of GPs has been increasing on account of the abuse of abdominal imaging methods, affecting approximately 4%–10% of adults worldwide (1, 2). Clinically, most gallbladder polyps are benign, and only a minority are malignant polyps. Unfortunately, the prognosis and clinical management of them are quite different (3–5). Thus, it is crucial to differentiate benign and malignant GPs preoperatively.

Recently, predictions of malignant GPs have been reported based on the features of patients and GPs. However, it has been proven difficult to differentiate between benign and malignant GPs relying on these features (6–8). Radiomics is an emerging method whose final goal is to dig up the existing medical images that we can get the high-dimensional information, hence aiding in clinical decision-making. In clinical practice, contrast-enhanced computed tomography (CECT) is in common use for GPs, benefited to evaluate the relationship of the tumor and surrounding tissues and consequently accurately diagnose GPs (9). Therefore, a tool for the early identification of malignant GPs is developed through this research.

## Materials and Methods

### Patient Selection

The study ultimately included 195 patients with gallbladder polypoid lesions which were ≥10 mm and proven by pathology during April 2014 to May 2021. The inclusion criteria included the following (1) patients who underwent surgical treatment and were diagnosed, confirmed pathologically; (2) the maximum diameter of GPs ≥10 mm; and (3) CECT performed in all patients within 1 month prior to the operation. The exclusion criteria included the following: (1) patients had undergone some operation or treatment before surgery including radio-chemotherapy and percutaneous transhepatic gallbladder drainage; (2) the lesion had invaded the surrounding tissues obviously; and (3) the GPs could not be displayed clearly for the gallbladder wall edema accompanied by a large amount of inflammatory exudate due to acute cholecystitis and respiratory artifacts. All the patients were randomly divided into a training cohort and a testing cohort in the ratio of 7:3.

### Clinical Feature

Clinical characters of patients and CT imaging features, measured by experienced radiologists, were collected and recorded from electronic medical records, retrospectively. The cutoff points of age were confirmed, and patients were divided into two groups based on the principle of maximum Youden’s index: patients who were younger than 56 years and not. If any of the gallbladder diseases symptoms existed, such as upper abdominal pain, nausea and vomiting, cutaneous, or sclera icterus, they were recorded as positive. The diameter of the lesion was recorded at the horizontal slice of the largest size of the lesion. In addition, the GPs were divided by the gallbladder anatomical location strictly, which was defined as gallbladder neck, fundus, or body. The base that means the basal morphology of GPs was divided into sessile and pedunculated for the angle between the basement mucosa and protuberance of the GPs with reference to Yamada’s classification where the sessile lesions refer to the angle >90° while the pedunculated lesions are defined as angle <90° (10). It would be defined as multiple if there were more than one lesion, and the lesion which had the largest diameter was considered as the target lesion. Each parameter was compared between the benign and malignant gallbladder polypoid lesion groups with univariate correlation analysis in the training cohort. Thereafter, the parameters that associated with the benignity and malignancy of the GPs were identified by a multivariate logistic regression analysis.

### Imaging

Three CT scanners were included in this study, namely, Somatom Definition Flash CT (Siemens Healthineers, Erlangen, Germany), Aquilion ONE CT (Toshiba Corporation, Tokyo, Japan), and BrillianceICT (Royal Philips Electronics, Amsterdam, Netherlands). The most recent record will be selected if the patient has multiple CECT examination records. All patients were first given a plain scan in a conventional supine position, then an enhanced scan. Arterial phase, portal venous phase, and delayed phase were performed at 25 to 30 s, 60 to 70 s, and 160 to 180 s after the injection of a non-ionic contrast agent. In addition, the whole original medical image is resampled to the same voxel spacing by the linear interpolation algorithm, and the differences of scanning parameters in different scanner modes are eliminated. The new data points in the original image are reconstructed within the range of known data points.

### Segmentation

The resampled sequences including arterial-phase, portal-phase, and delayed-phase CT images were imported to segment a structure software application called ITK-SNAP (http://www.itksnap.org, version 3.8.0), and the volume-of-interest (VOI) segmentation was manually delineated by a doctor with the years of radiology experience without seeing the patient’s clinical information or pathological diagnosis. Then, the delineated segmentations were reviewed carefully by a senior doctor who has 30 years of radiology experience.

### Radiomics Feature Extraction and Selection

“PyRadiomics,” an open-source package for standardizing the extraction of radiomics data (https://github.com/Radiomics/pyradiomics), was used to extract 107 radiomics features from each phase of preprocessed sequence CT image and the segmented VOI. The extracted features can be classified into seven categories including 14 Shape-based features, 18 First-Order Statistics features, 24 Gray-Level Co-occurrence Matrix features, 16 Gray-Level Run Length Matrix features, 16 Gray-Level Size Zone Matrix features, 14 Gray-Level Dependence Matrix features, and 5 Neighboring Gray Tone Difference Matrix features. All the features were standardized by the following formula: features = (f − µ)/std. First, Pearson correlation analysis was performed to identify the redundant features. Features with the mean absolute correlation higher than 0.8 were considered redundant and would be randomly eliminated by a high correlation filter, leaving only one. Then, the least absolute shrinkage and selection operator (LASSO) method with tenfold cross-validation was used to iteratively screen the most significant features in the training cohort until the feature coefficients were not zero. Rad-score was calculated based on these features by the formula shown as follows:


$$Rad-score=\beta_{0}+\beta_{1}x\;(f_{1}-\mu_{1})/std_{1}+\beta_{2}x\;(f_{2}-\mu_{2})/std_{2}+\cdots \beta_{n}x\;(f_{n}-\mu_{n})/std_{n}$$

f = {*f_i_*, i = 1, 2, ···, n} indicates the selected radiomics features; µ = {µ*_i_*, i = 1, 2, ···, n}

and std = {std*_i_*, i = 1, 2, ···, n} indicates the mean value and the standard deviation of each feature and β = {β*_i_*, i = 0, 1, ···, n} indicates the LASSO regression coefficient.

### Nomogram Building and Validation

**Figure 1:**
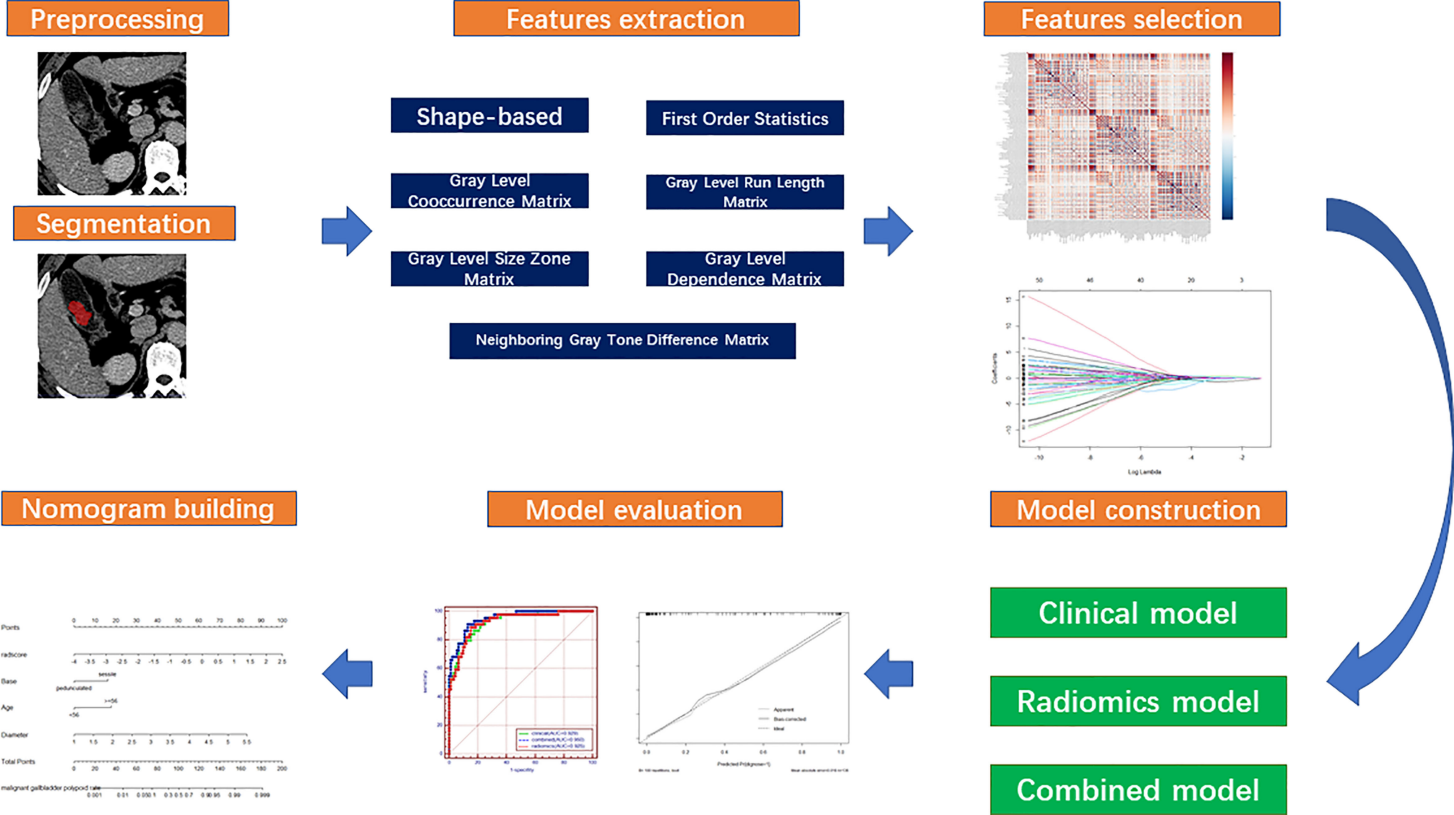
Study workflow.

After the clinical and radiomics significant parameters were identified in the training cohort, three models could be constructed in the training cohort with clinical features, radiomics features, or both of them, respectively. The receiver operating characteristic (ROC) curve and calibration curve were used to compare and evaluate the predictive ability of the models for GP benignity and malignancy in both the training and testing cohorts. Then, the area under the curve (AUC) was calculated and the cutoff value in the training cohort based on the maximum Youden’s index criterion was confirmed. On the same cutoff value, the sensitivity and specificity were achieved in both the training and testing cohorts. After assessment, the most robust model would be used to construct a nomogram. The whole process is shown in **Figure 1**.

### Statistical Analysis

Continuous data were expressed using the mean ± standard deviation (SD) or median [interquartile range (IQR) as appropriate. Continuous variables were analyzed using Student’s t-test or Mann–Whitney U test appropriately. Categorical variables were analyzed using the *x*^2^ test or Fisher exact test. All statistical analyses were completed by R language software (version 4.0.5). The packages named “ResourceSelection,” “PredictABEL,” “pROC,” “rms,” “glmnet,” “RROC,” “Hmisc,” and “rmda” were used.

## Results

### Patient Characteristics

A total of 195 patients were included in the study, including 136 in the training cohort and 59 in the testing cohort. In the training cohort, 92 patients with benign and 44 patients with malignant cystic polypoid lesions were enrolled, respectively. In the testing cohort, 40 patients have benign and 19 patients have malignant gallbladder polypoid lesions. The characteristics of patients in the training cohort are detailed in **Table 1**. The statistical meaningful characteristics were identified as significant with p < 0.1 by univariate analysis, among whom three characteristics were selected for p < 0.01 in multivariate logistic regression analysis by entering methods including age (odds ratio (OR) = 8.28, p = 0.003), base (OR = 6.96, p = 0.006), and diameter (OR = 14.68, p < 0.001) (**Table 2**). Taking three factors above as independent variables, a logistic regression model was constructed and evaluated. The sensitivity and specificity were 0.773, 0.935 in the training cohort and 0.737, 0.950 in the testing cohort, respectively, and the AUC was 0.929, 0.943, respectively.

**Table 1:** Demographics and clinical characteristics.

Parameters	Level	Overall	Benign	Malignant	p
		136	92	44	
Age (%)	<56	62 (45.6)	54 (58.7)	8 (18.2)	<0.001
	>=56	74 (54.4)	38 (41.3)	36 (81.8)
Sex (%)	female	89 (65.4)	61 (66.3)	28 (63.6)	0.91
	male	47 (34.6)	31 (33.7)	16 (36.4)
Diabetes (%)	absent	115 (84.6)	79 (85.9)	36 (81.8)	0.72
	present	21 (15.4)	13 (14.1)	8 (18.2)
Hypertension (%)	absent	103 (75.7)	77 (83.7)	26 (59.1)	0.004
	present	33 (24.3)	15 (16.3)	18 (40.9)
BMI (kg/m^2^)		25.95 [22.17, 30.10]	25.05 [21.87, 30.57]	27.55 [22.90, 29.70]	0.421
Symptoms (%)	absent	91 (66.9)	64 (69.6)	27 (61.4)	0.45
	present	45 (33.1)	28 (30.4)	17 (38.6)
CA199 (%)	absent	108 (79.4)	75 (81.5)	33 (75.0)	0.514
	present	28 (20.6)	17 (18.5)	11 (25.0)
CA125 (%)	absent	135 (99.3)	91 (98.9)	44 (100.0)	1
	present	1 (0.7)	1 (1.1)	0 (0.0)
AFP (%)	absent	128 (94.1)	85 (92.4)	43 (97.7)	0.437
	present	8 (5.9)	7 (7.6)	1 (2.3)
CEA (%)	absent	110 (80.9)	73 (79.3)	37 (84.1)	0.671
	present	26 (19.1)	19 (20.7)	7 (15.9)
RBC (10^12^/L)		4.57 (0.50)	4.62 (0.49)	4.48 (0.51)	0.126
HGB (g/L)		137.83 (15.98)	138.35 (16.32)	136.75 (15.38)	0.587
PLT (10^9^/L)		227.50 [197.50, 254.25]	229.00 [202.75, 257.50]	210.50 [190.25, 239.75]	0.078
INR		1.00 [1.00, 1.02]	1.00 [1.00, 1.02]	1.00 [1.00, 1.02]	0.493
WBC (10^9^/L)		5.67 [4.67, 7.02]	5.74 [4.64, 6.61]	5.46 [4.75, 7.37]	0.559
NE (10^9^/L)		3.16 [2.54, 3.97]	3.14 [2.55, 3.97]	3.23 [2.48, 4.69]	0.614
LY (10^9^/L)		1.92 [1.55, 2.35]	1.93 [1.61, 2.33]	1.75 [1.50, 2.38]	0.462
ALB (g/L)		40.69 (4.00)	41.37 (3.98)	39.27 (3.68)	0.004
ALT (U/L)		17.50 [12.00, 24.25]	17.50 [12.00, 24.00]	18.00 [12.00, 27.75]	0.622
DBIL (μmol/L)		3.10 [2.50, 4.70]	3.10 [2.48, 4.82]	3.10 [2.58, 4.43]	0.961
TBIL (μmol/L)		11.30 [8.70, 15.45]	11.70 [9.15, 15.93]	10.50 [8.30, 13.35]	0.35
Location (%)	Bottom/body	124 (91.2)	88 (95.7)	36 (81.8)	0.019
	Neck	12 (8.8)	4 (4.3)	8 (18.2)
Number (%)	Single	92 (67.6)	58 (63.0)	34 (77.3)	0.143
	Multiple	44 (32.4)	34 (37.0)	10 (22.7)
Base (%)	Pedunculated	47 (34.6)	41 (44.6)	6 (13.6)	0.001
	sessile	89 (65.4)	51 (55.4)	38 (86.4)
Diameter (cm)		1.60 [1.19, 2.30]	1.29 [1.07, 1.73]	2.43 [1.95, 3.35]	<0.001
Stones (%)	Absent	115 (84.6)	78 (84.8)	37 (84.1)	1
	Present	21 (15.4)	14 (15.2)	7 (15.9)

**Table 2:** Multivariate analysis of risk factors related with malignant GPs.

Parameters		Odds ratio	95% CI	p
Age	<56 vs. ≥56	8.28	2.05-33.46	0.003
Base	Pedunculated vs. sessile	6.96	1.77-27.46	0.006
diameter		14.68	4.38-49.16	<0.001

### Radiomics Features

**Figure 2:**
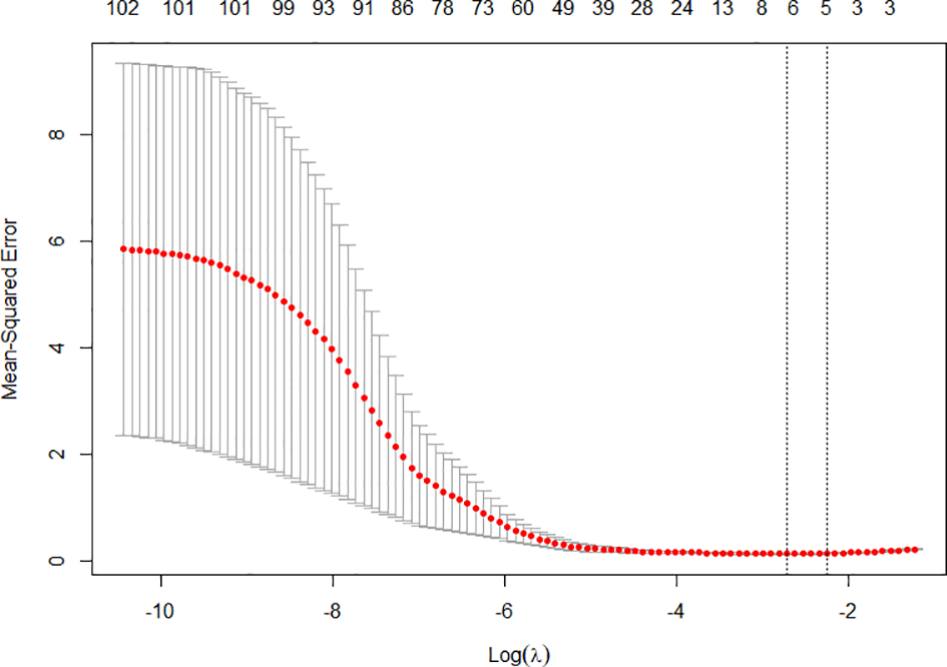
The tenfold cross-validation was repeated 100 times to generate the optimal value in the LASSO model. Six non-zero coefficients were chosen at the standard of lambda that gave the minimum binomial deviance.

After importing the original images and VOI segmentation files into the Pyradiomics package, 321 radiomics features of each patient were extracted and then normalized. Subsequently, Pearson’s correlation coefficients of all 321 radiological features for each patient and highly correlated features were randomly excluded, after which a total of 104 radiomics features remained. Six features with non-zero coefficients were finally filtrated by the LASSO logistic regression (**Figure 2**). Taking 6 radiomics factors into the radiomics model, Rad-score was calculated in the training cohort and testing cohort. The sensitivity and specificity were 0.886, 0.848 in the training cohort and 0.737, 0.925 in the testing cohort, respectively, and the AUC was 0.925, 0.920, respectively.

### Development, Performance, and Validation of the Combined Model

**Figure 3:**
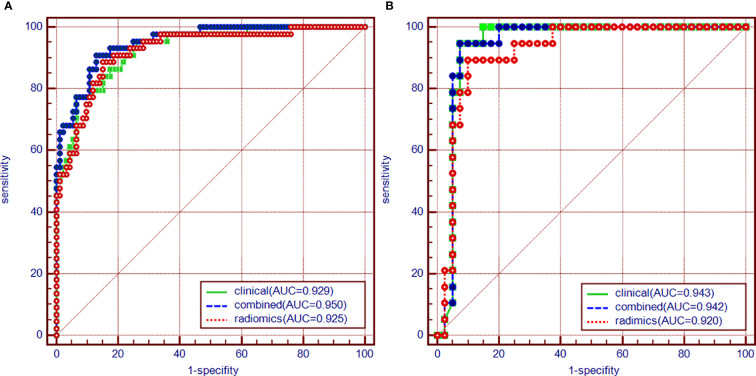
**(A)** Diagnostic efficiency of 3 models using ROC analysis in the training cohort. **(B)** Diagnostic efficiency of 3 models using ROC analysis in the testing cohort.

**Figure 4:**
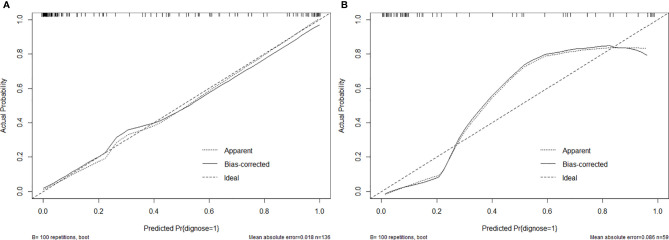
**(A)** Calibration curves of combined model in the training cohort. **(B)** Calibration curves of combined model in the testing cohort.

As aforementioned, we incorporated clinical features in conjunction with radiomics signatures into the multivariate logistic regression in the training cohort and obtained the combined logistic regression model. The sensitivity and specificity were 0.909, 0.870 in the training cohort and 0.842, 0.925 in the testing cohort, which performed more equally and appropriately as a screening model corresponding to other two models, and the AUC was 0.950, 0.942 (**Figures 3A, B**), respectively. Aside this, the calibration curves of the combined model in both training cohort and testing cohort showed that the discrete experimental lines were almost overlapping with or close to the diagonal line (**Figures 4A, B**), which indicated that the calibration of the combined model in identifying the benignity and malignancy of GPs was high. Moreover, the Hosmer–Lemeshow test yielded non-significant p values, 0.824 in the training cohort and 1.000 in the testing cohort, which also showed good calibration power.

## Discussion

Gallbladder lesions are broadly divided into wall thickening (GWT) and polypoid lesions (GPs) according to the morphology performance in imaging modalities that GWTs should be determined as wall thickening of 4 mm or more, while GPs are defined as focal elevation or protrusions that are distinguishable from the surrounding mucosa including early gallbladder cancer and neoplastic and non-neoplastic polyps (11, 12). For instance, the most common type of non-neoplastic polyp is cholesterol polyp, which accounts for about 60% of gallbladder polyps and tends to remain benign. Adenomas are truly neoplastic polyps with a definite potential to develop into a malignant state. Unfortunately, benign and malignant gallbladder polyps are difficult to distinguish because of their similar morphology, and there are currently no reliable predictive biomarkers for the diagnosis of GPs larger than 10 mm (13–15). At present, it is well accepted and recommended that, when polyps are larger than 10 mm, cholecystectomy should be performed because gallbladder polyps that are large-sized (≥10 mm) or rapidly growing must be regarded as potentially malignant (16–19). About the choice of initial surgical methods for GPs, laparoscopic cholecystectomy is recommended for GPs larger than 10 mm unless the malignant one is highly preoperative suspected without taking account of any other factors that might interfere with surgery, while the final approach of surgery is determined by intraoperative frozen sections and postoperative histopathology. Although the most definite surgical approach for malignant GPs is unsettled, it is widely recognized that open cholecystectomy with partial liver and lymph node resection when necessary or laparoscopic cholecystectomy is appropriate to achieve better prognosis according to invasive GPs or not. Therefore, it is important to preoperatively identify malignant GPs based on which can we take the proper surgical techniques such as avoidance of gallbladder perforation and bile spillage, use of a protective bag for specimen extraction, and intraoperative frozen sections or open cholecystectomy (13, 20, 21). Actually, there was only 0.690 of sensitivity in clinical preoperative diagnosis at our database (**Table 3**). Considering the moderate diagnostic accuracy, we developed and validated a nomogram incorporating clinical and radiomics features for individualized preoperative prediction and differentiation of benign and malignant GPs. The results showed that the study provided a prediction tool by which patients with gallbladder polyps ≥10 mm in size can be identified before surgery and had a favorable discrimination and calibration.

**Table 3:** The sensitivity and specificity of clinical diagnosis for malignant gallbladder polypoid-lesions were 0.6190 and 0.8939, respectively.

Clinical diagnosis		

Pathology diagnosis	Benign	Malignant
**Benign**	118	14
**Malignant**	24	39

For the selection of clinical characteristics and imaging features, previous studies have confirmed that several clinical risk factors were closely related to the benignity and malignancy of the GPs (7, 13). Similarly, we found that age, base, and diameter were significantly associated with the benignity and malignancy of the GPs in this study. In total, 26 candidate features were reduced to 3 features that influenced the benignity and malignancy of the GPs as independent factors after the univariate correlation analysis and multivariate logical regression in the training cohort. Actually, the presence of gallstones appears to be a risk factor for malignancy of GPs in previous studies but not ours. One possible contributing factor was that the increased risk caused by gallstones is most likely attributable to greater local epithelial irritation and chronic inflammation leading to dysplasia, which were presented with GWTs and excluded from our study.

In addition, traditional radiographic diagnosis by visual observation is usually limited by human visual perception, while radiomics was a useful tool, which enables quantification of diseases by extracting information that cannot be directly recognized by the human brain from images and ultimately assists the surgeon especially in diagnosis and efficacy prediction (6, 8, 11). For the achievement of the radiomics signature from image, ultrasonography (US) is one of the most effective screening methods used for assessment of GPs. The diagnostic performance of US can be further improved by the use of high-resolution US, contrast-enhanced US, and endoscopic US. Yuan et al. showed that contrast-enhanced US is preferred over CT for the diagnosis of neoplastic and non-neoplastic GPs; Andrea et al. reported the use of contrast-enhanced endoscopic US for the characterization of mural nodules within pancreatic cystic neoplasms; and Antonio et al. described a method of contrast-enhanced harmonic endoscopic ultrasound-guided fine-needle aspiration versus standard fine-needle aspiration in pancreatic masses (11, 22–24). However, we noted the limitation of ultrasonography (US) that the slices they selected in two-dimensional imaging may not cover the cancerous lesions area and the sensitivity and accuracy of US are highly dependent on the diagnostic skill of sonographers. Magnetic resonance imaging based on high b-value diffusion-weighted imaging has been applied as a non-invasive modality in distinguishing between benign and malignant GPs, but the sensitivity and specificity of magnetic resonance imaging are unsatisfying owing to the “T2 shine-through” effect (6, 13). Yet, CECT is a widespread used modality and is adopted most frequently to distinguish between benign and malignant gallbladder polypoid lesions, and it was adopted on a single phase by previous investigations (10). The potential of radiomics to predict the characteristics of tumors, among whom CT-based radiomics has been widely applied in liver, lung, and pancreatic tumors, has been demonstrated (25–27). However, there have been a few reports on CECT-based radiomics in predicting benign and malignant GPs. To the best of our knowledge, this study is the first attempt to provide a comprehensive analysis of the benignity and malignancy of the GPs involving three phases of CECT. In addition, High Correlation filter was used to eliminate one radiomics feature randomly which was considered highly correlated with another from the same or different phases of the same patient. The least absolute shrinkage and selection operator (LASSO) method with tenfold cross-validation was used for subsequent feature selection to avoid overfitting. Therefore, 321 candidate radiomics features were narrowed down to 6 potential predictors by High Correlation filter and LASSO method.

**Figure 5:**
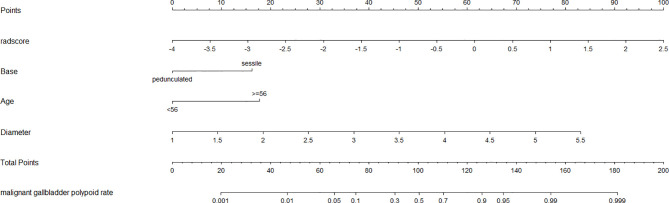
The developed nomogram based on combined model.

A clinical model that included 3 independent factors was constructed, while a radiomics model that incorporated 6 radiomics features and a combined model with Rad-score calculated by the radiomics model as well as 3 clinical features in the clinical model were established. The performance of the combined model in ROC was significantly more excellent than other models in the training cohort, which was identified as the best model for its superior sensitivity and specificity in the testing cohort at the situation of the same cutoff value with the training cohort, although the AUC of the combined model was less than the clinical model in the testing cohort probably due to the small sample size, on which the model can have the potential to identify more malignant GPs in external data. A nomogram was depicted based on the combined model (**Figure 5**).

Clinically, for GP patients with difficulty in diagnosis, after adjusting the balance of extra economic costs of CECT and clinical benefit, our data suggest that patients at high risk of malignant GPs according to our combined model should be treated with proper surgical approaches.

## Conclusion

The proposed combined model can provide a novel approach to effectively evaluate benign and malignant GPs, which assist compensatorily in the preoperative decision-making of malignant risk of lesions ≥10 mm in size.

## Data Availability Statement

The raw data supporting the conclusions of this article will be made available by the authors, without undue reservation.

## Ethics Statement

This study was approved by the Institutional Review Board of The First Hospital of China Medical University and the requirement for informed consent was waived based on the nature of a retrospective study.

## Author Contributions

SH and YL collected and analyzed the data. SH and XL drafted the manuscript. XJ and BL were involved in manuscript reviewing. CZ and JZ designed the study. All authors contributed to the article and approved the submitted version.

## Conflict of Interest

The authors declare that the research was conducted in the absence of any commercial or financial relationships that could be construed as a potential conflict of interest.

## Publisher’s Note

All claims expressed in this article are solely those of the authors and do not necessarily represent those of their affiliated organizations, or those of the publisher, the editors and the reviewers. Any product that may be evaluated in this article, or claim that may be made by its manufacturer, is not guaranteed or endorsed by the publisher.

## References

[1] Choi Y Do J Seo S Lee S Oh H Min Y. Prevalence and Risk Factors of Gallbladder Polypoid Lesions in a Healthy Population. Yonsei Med J (2016) 57:1370–5. doi: 10.3349/ymj.2016.57.6.1370 PMC5011268 27593864

[2] Lin W Lin D Tai D Hsieh S Lin C Sheen I. Prevalence of and Risk Factors for Gallbladder Polyps Detected by Ultrasonography Among Healthy Chinese: Analysis of 34 669 Cases. J Gastroenterol Hepatol (2008) 23:965–9. doi: 10.1111/j.1440-1746.2007.05071.x 17725602

[3] Xu A Zhang Y Hu H Zhao G Cai J Huang A. Gallbladder Polypoid-Lesions: What Are They and How Should They be Treated? A Single-Center Experience Based on 1446 Cholecystectomy Patients. J Gastrointestinal Surg (2017) 21:1804–12. doi: 10.1007/s11605-017-3476-0 28695432

[4] Babu B Dennison A Garcea G. Management and Diagnosis of Gallbladder Polyps: A Systematic Review. Langenbecks Arch Surg (2015) 400:455–62. doi: 10.1007/s00423-015-1302-2 25910600

[5] Singh S Agarwal AK. Gallbladder Cancer: The Role of Laparoscopy and Radical Resection. Ann Surg (2009) 250:494–5; author reply 495. doi: 10.1097/SLA.0b013e3181b490d9 19730180

[6] Ogawa T Horaguchi J Fujita N Noda Y Kobayashi G Ito K. High B-Value Diffusion-Weighted Magnetic Resonance Imaging for Gallbladder Lesions: Differentiation Between Benignity and Malignancy. J Gastroenterol (2012) 47:1352–60. doi: 10.1007/s00535-012-0604-1 22576026

[7] Cha B Hwang J Lee S Kim J Cho J Kim H. Pre-Operative Factors That Can Predict Neoplastic Polypoid Lesions of the Gallbladder. World J Gastroenterol (2011) 17:2216–22. doi: 10.3748/wjg.v17.i17.2216 PMC3092874 21633532

[8] Zhou W Li G Ren L. Triphasic Dynamic Contrast-Enhanced Computed Tomography in the Differentiation of Benign and Malignant Gallbladder Polypoid Lesions. J Am Coll Surgeons (2017) 225:243–8. doi: 10.1016/j.jamcollsurg.2017.04.014 28455251

[9] Aerts H. The Potential of Radiomic-Based Phenotyping in Precision Medicine: A Review. JAMA Oncol (2016) 2:1636–42. doi: 10.1001/jamaoncol.2016.2631 27541161

[10] Yang X Liu Y Guo Y Chai R Niu M Xu K. Utility of Radiomics Based on Contrast-Enhanced CT and Clinical Data in the Differentiation of Benign and Malignant Gallbladder Polypoid Lesions. Abdominal Radiol (New York) (2020) 45:2449–58. doi: 10.1007/s00261-020-02461-2 32166337

[11] Okaniwa S. How Can We Manage Gallbladder Lesions by Transabdominal Ultrasound? Diagnostics (Basel) (2021) 11:784. doi: 10.3390/diagnostics11050784 38248044 PMC10814927

[12] Yu M Kim Y Park H Jung S. Benign Gallbladder Diseases: Imaging Techniques and Tips for Differentiating With Malignant Gallbladder Diseases. World J Gastroenterol (2020) 26:2967–86. doi: 10.3748/wjg.v26.i22.2967 PMC7304100 32587442

[13] Xu A Hu H. The Gallbladder Polypoid-Lesions Conundrum: Moving Forward With Controversy by Looking Back. Expert Rev Gastroenterol Hepatol (2017) 11:1071–80. doi: 10.1080/17474124.2017.1372188 28837358

[14] Zielinski M Atwell T Davis P Kendrick M Que FG. Comparison of Surgically Resected Polypoid Lesions of the Gallbladder to Their Pre-Operative Ultrasound Characteristics. J Gastrointest Surg (2009) 13:19–25. doi: 10.1007/s11605-008-0725-2 18972168

[15] Kim S Kim H Yang D Ryu J Won KY. Gallbladder Carcinoma: Causes of Misdiagnosis at CT. Clin Radiol (2016) 71:e96–109. doi: 10.1016/j.crad.2015.10.016 26602932

[16] Yang H Sun Y Wang Z. Polypoid Lesions of the Gallbladder: Diagnosis and Indications for Surgery. Br J Surg (1992) 79:227–9. doi: 10.1002/bjs.1800790312 1555088

[17] Gallahan W Conway J. Diagnosis and Management of Gallbladder Polyps. Gastroenterol Clinics North America (2010) 39:359–67, x. doi: 10.1016/j.gtc.2010.02.001 20478491

[18] Boulton R Adams D. Gallbladder Polyps: When to Wait and When to Act. Lancet (London England) (1997) 349:817. doi: 10.1016/S0140-6736(05)61744-8 9121250

[19] Mainprize K Gould S Gilbert J. Surgical Management of Polypoid Lesions of the Gallbladder. Br J Surg (2000) 87:414–7. doi: 10.1046/j.1365-2168.2000.01363.x 10759734

[20] Lundgren L Muszynska C Ros A Persson G Gimm O Andersson B. Management of Incidental Gallbladder Cancer in a National Cohort. Br J Surg (2019) 106:1216–27. doi: 10.1002/bjs.11205 31259388

[21] Papageorge M de Geus S Woods A Ng S Drake F Cassidy M. Undertreatment of Gallbladder Cancer: A Nationwide Analysis. Ann Surg Oncol (2021) 28:2949–57. doi: 10.1245/s10434-021-09607-6 33566241

[22] Yuan Z Liu X Li Q Zhang Y Zhao L Li F. Is Contrast-Enhanced Ultrasound Superior to Computed Tomography for Differential Diagnosis of Gallbladder Polyps? A Cross-Sectional Study. Front Oncol (2021) 11:657223. doi: 10.3389/fonc.2021.657223 34109116 PMC8181139

[23] Lisotti A Napoleon B Facciorusso A Cominardi A Crinò S Brighi N. Contrast-Enhanced EUS for the Characterization of Mural Nodules Within Pancreatic Cystic Neoplasms: Systematic Review and Meta-Analysis. Gastrointestinal Endoscopy (2021) 94(5):881–9.e5. doi: 10.1016/j.gie.2021.06.028 34217751

[24] Facciorusso A Mohan B Crinò S Ofosu A Ramai D Lisotti A. Contrast-Enhanced Harmonic Endoscopic Ultrasound-Guided Fine-Needle Aspiration Versus Standard Fine-Needle Aspiration in Pancreatic Masses: A Meta-Analysis. Expert Rev Gastroenterol Hepatol (2021) 15:821–8. doi: 10.1080/17474124.2021.1880893 33481633

[25] Zhang W Yang R Liang F Liu G Chen A Wu H. Prediction of Microvascular Invasion in Hepatocellular Carcinoma With a Multi-Disciplinary Team-Like Radiomics Fusion Model on Dynamic Contrast-Enhanced Computed Tomography. Front Oncol (2021) 11:660629. doi: 10.3389/fonc.2021.660629 33796471 PMC8008108

[26] Li Q He X Fan X Zhu C Lv J Luo T. Development and Validation of a Combined Model for Preoperative Prediction of Lymph Node Metastasis in Peripheral Lung Adenocarcinoma. Front Oncol (2021) 11:675877. doi: 10.3389/fonc.2021.675877 34109124 PMC8180898

[27] Palumbo D Mori M Prato F Crippa S Belfiori G Reni M. Prediction of Early Distant Recurrence in Upfront Resectable Pancreatic Adenocarcinoma: A Multidisciplinary, Machine Learning-Based Approach. Cancers (2021) 13:4938. doi: 10.3390/cancers13194938 34638421 PMC8508250

